# Sex differences in body composition and bone mineral density in phenylketonuria: A cross-sectional study

**DOI:** 10.1016/j.ymgmr.2018.01.004

**Published:** 2018-02-03

**Authors:** Bridget M. Stroup, Karen E. Hansen, Diane Krueger, Neil Binkley, Denise M. Ney

**Affiliations:** a Department of Nutritional Sciences, University of Wisconsin-Madison, Madison, WI, USA; b Department of Medicine, Divisions of Rheumatology and Endocrinology, University of Wisconsin School of Medicine and Public Health, Madison, WI, USA; c Department of Medicine, University of Wisconsin School of Medicine and Public Health, Madison, WI, USA; d Department of Medicine, Divisions of Endocrinology and Geriatrics, University of Wisconsin School of Medicine and Public Health, Madison, WI, USA

**Keywords:** Amino acid, Appendicular lean mass index, Glycomacropeptide, Medical food, Osteoporosis, Renal net acid, Trabecular bone score, Urinary calcium excretion, AA-MF, Amino acid medical foods, ALM, Appendicular lean mass, BMD, Bone mineral density, DXA, Dual-energy X-ray absorptiometry, GMP-MF, Glycomacropeptide medical foods, MF, Medical foods, PAH, Phenylalanine hydroxylase, PE, Protein equivalent, Phe, Phenylalanine, PKU, Phenylketonuria, PRAL, Potential renal acid load, RDN, Registered Dietitian Nutritionist, TBS, Trabecular bone score, Tyr, Tyrosine

## Abstract

**Background:**

Low bone mineral density (BMD) and subsequent skeletal fragility have emerged as a long-term complication of phenylketonuria (PKU).

**Objective:**

To determine if there are differences in BMD and body composition between male and female participants with PKU.

**Methods:**

From our randomized, crossover trial [1] of participants with early-treated PKU who consumed a low-phenylalanine (Phe) diet combined with amino acid medical foods (AA-MF) or glycomacropeptide medical foods (GMP-MF), a subset of 15 participants (6 males, 9 females, aged 15–50 y, 8 classical and 7 variant PKU) completed one dual energy X-ray absorptiometry (DXA) scan and 3-day food records after each dietary treatment. Participants reported lifelong compliance with AA-MF. In a crossover design, 8 participants (4 males, 4 females, aged 16–35 y) provided a 24-h urine collection after consuming AA-MF or GMP-MF for 1–3 weeks each.

**Results:**

Male participants had significantly lower mean total body BMD Z-scores (means ± SE, males = − 0.9 ± 0.4; females, 0.2 ± 0.3; *p* = 0.01) and tended to have lower mean L1–4 spine and total femur BMD Z-scores compared to female participants. Only 50% percent of male participants had total body BMD Z-scores above − 1.0 compared to 100% of females (*p* = 0.06). Total femur Z-scores were negatively correlated with intake of AA-MF (*r* = − 0.58; *p* = 0.048). Males tended to consume more grams of protein equivalents per day from AA-MF (means ± SE, males: 67 ± 6 g, females: 52 ± 4 g; *p* = 0.057). Males and females demonstrated similar urinary excretion of renal net acid, magnesium and sulfate; males showed a trend for higher urinary calcium excretion compared to females (means ± SE, males: 339 ± 75 mg/d, females: 228 ± 69 mg/d; *p* = 0.13). Females had a greater percentage of total fat mass compared to males (means ± SE, males: 24.5 ± 4.8%, females: 36.5 ± 2.5%; *p* = 0.047). Mean appendicular lean mass index was similar between males and females. Male participants had low-normal lean mass based on the appendicular lean mass index.

**Conclusions:**

Males with PKU have lower BMD compared with females with PKU that may be related to higher intake of AA-MF and greater calcium excretion. The trial was registered at www.clinicaltrials.gov as NCT01428258.

## Highlights

• Males with PKU showed lower total body BMD and tended to excrete more calcium in urine compared to females with PKU.
• Males with PKU had low-normal lean body mass and females with PKU had greater fat mass than males.
• Our data suggest that males with PKU may be at greater risk for osteoporosis than females with PKU.

## 1 Introduction

PKU (PKU; OMIM 261600) is an autosomal recessive genetic disease that results in a deficiency of phenylalanine hydroxylase (PAH; EC 1.14.16) to hydroxylate Phe to tyrosine (Tyr), using tetrahydrobiopterin as a cofactor [2]. Early identification of PKU with newborn screening and initiation of a low-Phe diet within the first weeks of life are essential to prevent severe cognitive impairment caused by the neurotoxicity of high Phe concentrations in the brain [3], [4]. Primary treatment for PKU involves lifelong adherence to a low-Phe diet, restricted in protein from natural foods, in combination with low-Phe amino acid medical foods (AA-MF) or glycomacropeptide medical foods (GMP-MF) to meet daily protein and micronutrient needs [4], [5].

Skeletal fragility, characterized by low bone mineral density (BMD) and increased fracture risk, is a long-term complication of PKU for which incidence, etiology and prevalence are poorly understood. Approximately 40–50% of adults and 33% of children with PKU, treated with AA-MF lifelong, sustain fragility fracture [6], [7]. Additionally, femora from PKU^enu2/enu2^ mice have lower BMD, and biomechanical analysis indicates that they fracture with less force than wild type littermate control mice [8]. However, it is unclear whether there are differences in indicators of bone health between males and females with PKU [9], [10], [11], [12], [13], [14], [15], [16], [17], [18], and often, comparisons for males and females are not pursued [6], [7], [19], [20], [21], [22], [23], [24], [25], [26].

We recently conducted a randomized, controlled crossover trial to investigate the safety and efficacy of Phe-free AA-MF and low-Phe GMP-MF in 30 participants with early treated PKU and concluded that GMP-MF did not significantly increase plasma Phe concentrations [1]. From this clinical trial, two sub-studies were conducted: 1) A cross-sectional study in which one dual energy X-ray absorptiometry (DXA) scan was obtained from 15 participants with PKU; 2) A crossover pilot study in which 24-h urine collections and food records were obtained from 8 participants with PKU consuming AA-MF and GMP-MF to determine the impact of dietary acid load on excretion of renal net acid and minerals [9]. In our recent crossover pilot study, we demonstrated that ingestion of high-acid AA-MF significantly increased urinary excretion of renal net acid, calcium, and magnesium and concluded that this may negatively affect bone health in PKU [9]. Unexpectedly, we identified that 2 of 8 participants (both males) had low BMD-for-age based on DXA. We hypothesize that higher intake of AA-MF needed to support an intense pubertal growth spurt, may increase urinary calcium excretion and reduce bone accretion in males with PKU. Our objective was to investigate whether there are differences in BMD and body composition between males and females with PKU.

## 2 Methods

### 2.1 Study design and protocol

As stated in the introduction, this manuscript presents data from two sub-studies of our previously reported randomized, controlled, crossover trial [1]. First, utilizing a cross-sectional study design, we obtained one whole-body DXA scan from 15 of 30 participants who completed our randomized, controlled, crossover trial where participants consumed their typical low-Phe diet in combination with average intake of 0.74–0.76 g protein equivalents/kg/day from AA-MF or Glytactin™ GMP-MF for 3-wk each [1]. Intakes of medical foods composed of primarily elemental amino acids are described as protein equivalents. Participants reported lifelong intake of AA-MF prior to the trial. Thus, DXA scans reflect intake with AA-MF.

Second, a crossover pilot study was conducted in 8 of 30 particpants with early treated PKU [9]. Participants consumed a low-Phe diet in combination with a Glytactin™ GMP-MF with a low potential renal acid load and an AA-MF with a high potential renal acid load for 1–3 weeks each. Participants provided one 24-h urine collection for each dietary treatment and two-three 24-h food records before and during the 24-h urine collection. Food records for all studies were analyzed using Food Processor SQL (version 10.12.0, ESHA) [1], [9].

Briefly, for the crossover pilot study, the nutrient intakes of the low-Phe diet with AA-MF and GMP-MF treatments were generally similar except for the dietary protein source of medical foods, such that amino acids were consumed with AA-MF and primarily intact protein with GMP-MF. Mean intakes of total energy (2266–2566 kcal/d), total protein (79–81 g/d), and protein equivalents from medical foods (55-57 g protein equivalents/d) were similar between dietary treatments [9]. Despite similar intakes of total calcium (1745–1898 mg/d) and magnesium (568–684 mg/d), participants excreted more urinary calcium and magnesium with AA-MF than GMP-MF [9]. Consistent with the greater potential renal acid load (mEq/d) with AA-MF compared to Glytactin™ GMP-MF (means ± SE, AA-MF, 39 ± 5; GMP-MF, − 43 ± 6; *p* < 0.0001), excretion of renal net acid was 3-fold high with ingestion of AA-MF compared with GMP-MF [9].

Inclusion criteria included PKU diagnosis that was early-treated with medical food, a current prescribed diet providing > 50% of daily protein needs from AA-MF, and enrollment or completion of our clinical trial at the Waisman Center site [1]. Classical and variant PKU were defined based on genotype and response to sapropterin dihydrochloride [1]. The University of Wisconsin-Madison Health Sciences review board approved the study protocol. All participants provided written informed consent. The trial was registered at www.clinicaltrials.gov as NCT01428258.

### 2.2 Clinical measurements

Bone mineral density (BMD) and body composition were measured using a single GE-Healthcare Lunar iDXA densitometer (Madison, WI, USA) [9]. DXA scans were obtained and analyzed using enCORE software version 13.31 or 13.6. Weight-adjusted BMD Z-scores were derived using the manufacturer's sex-specific normative database. Spine trabecular bone scores (TBS) were obtained using Medimaps Group TBS inSight software version 2.0.0.1 or 2.1.0.0. (Mérignac, France) [27]. The appendicular lean mass index (ALMI) was calculated as the sum of lean mass of arms and legs (kg) divided by height squared (m^2^) [28]. ALMI Z-scores were analyzed using enCORE software version 17.0 with the USA NHANES 1999–2004 reference population for participants over 20 years of age. Mean DXA parameters for male and female participants and the statistical comparisons evaluating differences related to sex and PKU genotype are herein reported for the first time. Detailed methods related to the analysis of the 24-h urine collections have been previously reported [9]. Potential renal acid load was calculated to predict dietary acid load from AA-MF, using the following equation: Potential renal acid load (mEq/d) = (2 × (0.00503 × mg Met/d)) + (2 × (0.0062 × mg Cys/d)) + (0.037 × mg phosphorus/d) + (0.0268 × mg chloride/d) − (0.021 × mg potassium/d) − (0.026 × mg magnesium/d) − (0.013 × mg calcium/d) − (0.0413 × mg sodium/d) [9], [29], [30].

### 2.3 Statistical analysis

All statistical analyses were performed using SAS version 9.4 and assumptions of normality and equal variance were tested. Most analyses used PROC MIXED (SAS Institute Inc.). Participant characteristics and DXA scan data were analyzed using ANOVA with effects for sex and genotype (classical or variant PKU). The Kruskal-Wallis test was used to test for differences due to diet or genotype, if data was skewed. Statistical significance was set at *p* < 0.05.

## 3 Results

### 3.1 Participants

Fifteen (6 males, 9 females) participated in this sub-study, including 12 adults (aged 19–50 y) and 3 adolescents (aged 15–17 y). Participant characteristics are summarized in Table 1. Of the 8 participants categorized with classical PKU, all were adults (4 males, 4 females). Of the 7 participants categorized with variant PKU, 4 were adults (1 male, 3 females) and 3 were adolescents (1 male, 2 females). Although we tended to have more females than males with variant PKU, BMD or BMD Z-scores were similar between participants with classical and variant PKU (Supplemental Table 1). Two participants (both female adolescents) used a consistent dose of sapropterin dihydrochloride throughout the study.

**Table 1:** Participant characteristics.

Variable	Males (*n* = 6)	Females (*n* = 9)
Age group		
Adults	5	7
Adolescents (15-17y)	1	2
Age, y	28 ± 7	29 ± 11
Genotype		
Classical PKU	4	4
Variant PKU	2	5
Sapropterin dihydrochloride use	0	2
BMI, kg/m^2^	24.6 ± 4.0	26.8 ± 5.8
Plasma Phe, μmol/L	753 ± 285	613 ± 268

Data are presented as *n* for categorical variables or means ± SD for continuous variables. Participant data for age and BMI were obtained at the time of DXA scan completion. Data on genotype, sapropterin dihydrochloride use and Phe were obtained at visit 1 of the previously reported clinical trial [26].

BMI, body mass index; Phe, phenylalanine; PKU, phenylketonuria.

### 3.2 Bone mineral density

**Fig. 1:**
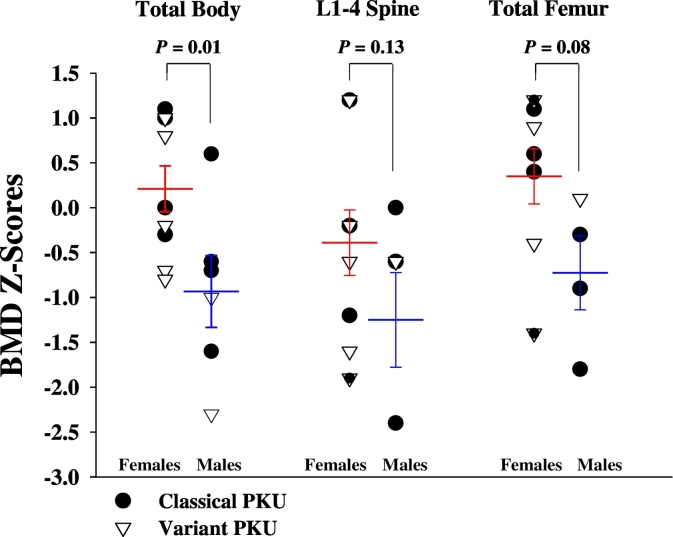
Comparison of BMD Z-scores of male and female participants with classical and variant PKU. Male participants had significantly lower total body BMD Z-Scores (*p* = 0.01; males, n = 6; females, n = 9) and tended to have lower L1–4 spine (*p* = 0.13; males, *n* = 6; females, *n* = 9) and total femur BMD Z-Scores (*p* = 0.08; males, *n* = 4; females, *n* = 8) compared to female participants. Values are means ± SE. BMD, bone mineral density; PKU, phenylketonuria.

Bone mineral density parameters are summarized in Table 2. Male participants had significantly lower mean total body BMD Z-scores (means ± SE: − 0.9 ± 0.4 vs. 0.2 ± 0.3, *p* = 0.01) and tended to have lower mean L1–4 spine and total femur BMD Z-scores compared to female participants (Fig. 1). Furthermore, only 50% of male participants had total body BMD Z-scores above − 1.0, compared to 100% of female participants (*p* = 0.06). Two of 15 participants were diagnosed with low BMD-for-age, i.e. a Z-score ≤ − 2.0 (1 adult male with classical PKU and 1 adult male with variant PKU). Of note, four additional participants (2 males, 2 females) had at least 1 BMD Z-score that was between − 1.0 and − 2.0 and one female participant had four BMD Z-scores between − 1.0 and − 2.0. There were no male versus female differences in BMD (g/cm^2^) for total body, L1–4 spine, total femur, femoral neck and femoral trochanter. BMD (g/cm^2^) and BMD Z-scores were similar in classical and variant PKU (Supplemental Table 1). Because of our small sample size and lack of age- and sex-matched controls, BMD Z-scores were used to compare differences due to sex and PKU genotype.

**Table 2:** Bone and body composition assessments in males and females with phenylketonuria^a^.

	Males		Females		*P*	
	n	Mean ± SE	n	Mean ± SE	Sex	gt
Total body						
Total fat mass, %	6	24.5 ± 4.8	9	36.5 ± 2.5	0.047	0.58
Total lean mass, kg	6	55.3 ± 2.8	9	41.9 ± 1.4	0.0008	0.53
ALM, kg	6	24.8 ± 1.5	9	18.0 ± 0.8	0.0002	0.58
ALMI, kg/m^2d^	6	7.99 ± 0.31	9	6.96 ± 0.35	0.07	0.78
ALMI Z-scores	5	− 0.04 ± 0.3	7	0.7 ± 0.3	0.17	0.46
BMD, g/cm^2^	6	1.099 ± 0.037	9	1.111 ± 0.029	0.60	0.23
Z-scores^b^	6	− 0.9 ± 0.4	9	0.2 ± 0.3	0.01	0.15
> − 1, n		3		9	0.06	–
Between − 1 and − 2, n		2		0		
Low for age (< − 2), n		1		0		
Spine L1-L4						
BMD, g/cm^2^	6	1.069 ± 0.057	9	1.139 ± 0.043	0.24	0.28
Z-scores	6	− 1.3 ± 0.5	9	− 0.4 ± 0.4	0.13	0.27
> − 1, n		4		6	1.00	–
Between − 1 and − 2, n		0		3		
Low for age (< − 2), n		2		0		
Trabecular bone score	6	1.37 ± 0.04	9	1.41 ± 0.02	0.46	0.52
Total femur^c^						
BMD, g/cm^2^	4	1.014 ± 0.049	8	1.029 ± 0.049	0.97	0.58
Z-scores^d^	4	− 0.7 ± 0.4	8	0.4 ± 0.3	0.08	0.86
> − 1, n		3		7	1.00	–
Between − 1 and − 2, n		1		1		
Low for age (< − 2), n		0		0		
Femoral neck						
BMD, g/cm^2^	4	0.996 ± 0.031	8	1.005 ± 0.031	0.94	0.69
Z-scores^d^	4	− 0.1 ± 0.2	8	− 0.7 ± 0.3	0.13	0.65
> − 1, n		3		7	1.00	–
Between − 1 and − 2, n		1		1		
Low for age (< − 2), n		0		0		
Femoral trochanter						
BMD, g/cm^2^	4	0.835 ± 0.039	8	0.829 ± 0.059	0.96	0.61
Z-scores^d^	4	− 1.1 ± 0.6	8	0.0 ± 0.3	0.16	0.63
> − 1, n		2		7	0.24	–
Between − 1 and − 2, n		1		1		
Low for age (< − 2), n		1		0		

ALM, appendicular lean mass; ALMI, appendicular lean mass index; BMD, bone mineral density; PKU, phenylketonuria.

a Values were obtained at the time of DXA scan completion, *n* = 15. Statistical analysis included ANOVA with main effects for sex and genotype (classical or variant PKU). Two of 15 participants were diagnosed with low BMD-for-age, based on Z-scores < − 2.0.

b One participant, whose Z-scores were included in this analysis, required T-scores for interpretation of DXA scan data due to post-menopausal status.

c BMD and Z-scores for femur-related DXA data represent an average for 11 of 12 subjects. Three participants have missing DXA data for the femur. Femur data for 1 participant is based on one femur due to presence of metal in the left hip.

d ALMI or ALM/ht^2^ was calculated as the sum of lean mass of arms & legs (kg)/height^2^ (m^2^) [28].

Participants with classical and variant PKU reported lifelong compliance with AA-MF and similar protein intake from medical foods during the course of our previously reported study (means ± SE, g protein equivalents from medical foods, classical PKU = 56 ± 3; variant PKU = 46 ± 3; *p* = 0.13) [9]. Male participants tended to have higher intakes of protein equivalents from AA-MF and greater urinary calcium excretion compared to female participants, in spite of similar intake of protein equivalents from AA-MF when adjusted for body weight or total lean mass.

**Fig. 2:**
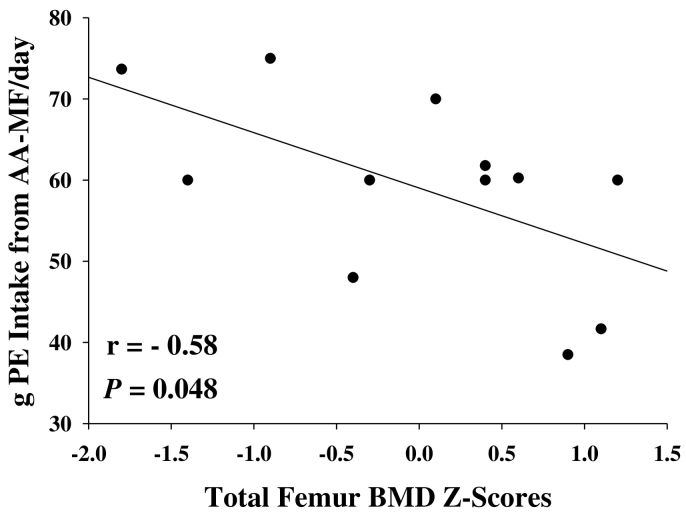
Total femur BMD Z-scores and intake of PE from AA-MF were negatively correlated (r = − 0.58, *p* = 0.048) based on 13 participants with PKU. AA-MF, amino acid medical foods; BMD, bone mineral density; PE, protein equivalent; PKU, phenylketonuria.

We utilized correlation coefficients to test the hypothesis that intake of AA-MF may negatively impact bone. Total femur Z-scores were negatively correlated with intake of AA-MF (*r* = − 0.58; *p* = 0.048), but not spine L1–4 Z-scores (*r* = − 0.17; *p* = 0.55) or total body Z-scores (*r* = − 0.42; *p* = 0.12) (Fig. 2). Due to the cross-sectional design and small sample size of this study, etiology for the significant correlation between intake of AA-MF and total femur Z-scores, but not spine L1–4 or total body Z-scores, is not clear. Regardless, this finding supports evidence from our previous study that the high dietary acid load of AA-MF increases urinary excretion of renal net acid, calcium, and magnesium and may increase bone resorption [9]. However, in the current study, we did not find significant differences between males and females in potential renal acid load from AA-MF nor urinary excretion of renal acid, magnesium, and sulfate (Table 3). Nonetheless, males tended to excrete more calcium than females, which could potentially have a negative impact on bone mineral density over time.

**Table 3:** Daily dietary intakes and urine excretion in male and female participants with phenylketonuria consuming AA-MF and GMP-MF^a^.

	Males	Females	*P*-values	
	Mean ± SE	Mean ± SE	Sex	gt
Diet				
PRAL from AA-MF, mEq	46 ± 8	33 ± 8	0.41	0.28
g PE from AA-MF	67 ± 6	52 ± 4	0.057	0.09
g PE from AA-MF/kg body weight	0.89 ± 0.09	0.77 ± 0.08	0.46	0.21
g PE from AA-MF/kg lean mass	1.20 ± 0.08	1.24 ± 0.10	0.54	0.14
Urine				
RNAE, mEq	9 ± 20	18 ± 14	0.74	0.67
Calcium, mg	339 ± 75	228 ± 69	0.15	0.59
Magnesium, mg	231 ± 33	208 ± 39	0.37^b^	0.60^b^
Sulfate, mEq	23 ± 5	23 ± 5	0.51	1.00

AA-MF, amino acid medical foods; PE, protein equivalent; PRAL, potential renal acid load; PKU, phenylketonuria; RNAE, renal net acid excretion.

a Dietary intakes of PE and PRAL from AA-MF are based on 3-day food records, n = 15. Body weight and lean mass estimates were obtained with a DXA scan. Urine excretion parameters are based on two 24-h urine collections collected from participants with AA-MF and GMP-MF treatments, *n* = 8. Statistical analysis included ANOVA with main effects for sex and genotype (classical or variant PKU).

b Kruskal-Wallis test used when data were skewed.

### 3.3 Body composition

Body composition assessments are summarized in Table 2. Consistent with the general population, female participants had significantly greater total fat mass compared to male participants (means ± SE, females, 36.5 ± 2.5%; males, 24.5 ± 4.8, *p* = 0.047). Three of 6 male participants and 4 of 9 female participants had excess fat mass, defined as fat mass ≥ 25% in males and ≥ 35% in females per the World Health Organization [31]. As expected, males had significantly more total lean mass and ALM. Unlike the general population where men have greater ALMI (also known as ALM/ht^2^), there was no significant difference in the ALMI and also no difference in ALMI Z-scores between male and female participants. Notably, the mean ALMI for our young male participants was close to the cut point of ≤ 7.26 kg/m^2^, a suggested cut-off to identify sarcopenia in older men [28], [32]. In summary, male participants had low-normal lean mass and female participants had significantly greater fat mass.

## 4 Discussion

The prevalence of low BMD-for-age in PKU, defined as a Z-score ≤ − 2.0, is high, reportedly affecting approximately 20% of individuals with PKU in comparison to 2% of a general population [7], [33], [34]. We have previously reported that AA-MF provide a high dietary acid load that increases urinary excretion of renal net acid and bone-related minerals (calcium and magnesium), which may contribute to skeletal fragility in PKU [9]. To build on this, in this study, we observed significantly lower BMD Z-scores for total body and trends for lower BMD Z-scores for L1–4 spine and total femur in male participants compared to females. Although we found no significant differences between males and females in the urinary excretion of renal net acid, magnesium or sulfate, males tended to excrete more calcium compared to females. This finding is important given that we have previously shown that urinary calcium excretion is negatively correlated with L1–4 spine BMD in 8 participants with lifelong compliance with high-acid AA-MF [9].

Conflicting evidence exists as to whether there are differences in BMD or BMD Z-scores between males and females with PKU; only two studies, both cross-sectional in design, have reported such data [10], [11]. In 28 participants with PKU (11 males/17 females, aged 10–33 y), Pérez-Dueñas et al. found that male participants had significantly lower L1–4 BMD Z-scores than females (means ± SE, males = − 1.58 ± 0.34; females = − 0.57 ± 0.19; *p* < 0.05) [11]. Similarly, in 88 participants with PKU (34 males/53 females, aged 19 ± 11y), Coakley et al. found that male participants tended to have lower total body BMD Z-scores than females (means ± SD, males = − 0.58 ± 1.06; females = − 0.17 ± 1.03; *p* = 0.07) [10]. By contrast, five studies reported no differences in BMD Z-scores between males and females with PKU, although data and/or statistics were not shown [13], [14], [15], [16], [18]. Moreover, many studies do not report data separately for sex or perform statistical analyses to test for differences due to sex [6], [7], [19], [22], [24], [25]. Such conflicting evidence highlights the importance to report indicators of bone health in PKU by sex.

Individuals with PKU and others who consume the majority of their dietary protein from amino acids instead of intact dietary proteins are estimated to have 20–40% higher protein requirements [4], [5], due to evidence of lower protein retention and protein synthesis with amino acids compared with intact protein [35], [36], [37]. Adequate intake of dietary protein or protein equivalents is important to optimize growth, especially given evidence of low-normal lean mass and excess fat mass observed in our adolescent and adult PKU participants. The observation that our young male participants had a mean ALMI that was similar to our female participants and was close to a suggested cut-off point to identify sarcopenia in older men was surprising. Although the etiology of this observation is unclear, possible explanations may include increased protein degradation compared to protein synthesis related to lifestyle and differences in protein metabolism associated with the PKU genotype or hormones [35], [36], [37]. Life style factors that might impact muscle may include suboptimal physical activity, limited intake of intact protein from natural foods, and consumption of elemental amino acids as opposed to intact protein from medical foods [32], [35], [36], [37].

While it is tempting to recommend higher intake of protein equivalents from AA-MF for individuals with PKU, our data suggest negative effects on bone health when AA-MF with a high potential renal acid load provides the primary source of protein [9]. We observed a negative correlation between protein intake from AA-MF and total femur Z-scores similar to that reported in a larger data set [10]. Moreover, our male participants showed lower BMD Z-scores, tended to consume more protein equivalents from AA-MF, and excreted more calcium compared to female participants. Given the limited understanding of bone health in PKU, a conservative approach is to encourage adequate intake of AA-MF or GMP-MF that have a low potential renal acid load in combination with weight-bearing exercise to support bone remodeling [6], [9], [38]. Acquisition of lean mass to promote bone strength is important given recent evidence of reduced bone strength in relation to muscle force in patients with PKU assessed with peripheral quantitative computed tomography [6].

In conclusion, our data demonstrate that males with PKU have lower total body BMD Z-scores and may be at greater risk for osteoporosis than females with PKU. We hypothesize that the lower total body BMD Z-scores found in our male participants may be related to low-normal lean mass and/or higher intakes of AA-MF with a correspondingly greater loss of urinary calcium. The study supports existing Genetic Metabolic Dietitian International Guidelines that recommend periodic measurement of BMD in individuals with PKU [4], [5]. Although limited by a small sample size (as is often the case in research studies in patients with rare diseases) and use of whole body DXA scans, our study suggests that AA-MF had a negative effect on BMD. Furthermore, our findings highlight the need to separately report indicators of bone health for males and females with PKU. Additional research, particularly interventional studies, to investigate the impact of AA-MF in the acquisition of bone mass in individuals with PKU are needed, especially in males.

The following is the supplementary data related to this article.

Supplementary Table 1 Bone and body composition assessments in participants with classical and variant phenylketonuria. Supplementary Table 1

## Ethical approval

The University of Wisconsin-Madison Health Sciences review board approved the study protocol. All subjects provided written informed consent. The trial was registerd at www.clinicaltrials.gov as NCT01428258.

## Competing interests

Denise M. Ney is a co-inventor on U.S. Patent 8,604,168 B2, “Glycomacropeptide Medical Foods for Nutritional Management of Phenylketonuria and other Metabolic Disorders,” which is held by the Wisconsin Alumni Research Foundation and licensed to Cambrooke Therapeutics, LLC. Denise M. Ney is a consultant to Arla Foods Ingredients and Agropur. Neil Binkley has received research support from General Electric Healthcare and is a consultant for Nestle. Bridget M. Stroup, Karen E. Hansen, and Sangita G. Murali have no conflicts of interest to declare.

## Funding

This work was supported by Department of Health and Human Services grants R01 FD003711 from the FDA Office of Orphan Product Development to Ney, P30-HD-03352, and by the Clinical and Translational Science Award (CTSA) program, through the NIH National Center for Advancing Translational Sciences (NCATS), grant UL1TR000427. Cambrooke Therapeutics, Inc. donated the GMP medical foods used in this study, but was not involved in the design or conduct of the study or in the collection, analysis or interpretation of the data.
