# Supplementary material for: Sex differences in body composition and bone mineral density in phenylketonuria: A cross-sectional study
Source: Mol Genet Metab Rep. 2018 Feb 3;15:30–5. doi: 10.1016/j.ymgmr.2018.01.004 (PMC6047464; doi:10.1016/j.ymgmr.2018.01.004)
Supplement: Supplementary Table 1 — Bone and body composition assessments in participants with classical and variant phenylketonuria. [file mmc1.docx]

| SUPPLEMENTAL TABLE 1. Bone and body composition assessments in participants with classical and variant phenylketonuria^1^ | | | | | | | | | | |
| --- | --- | --- | --- | --- | --- | --- | --- | --- | --- | --- |
|  | Classical PKU | | | |  | Variant PKU | | | | *P* |
|  | n | mean | ± | SE |  | n | mean | ± | SE | gt |
| **Total Body** |  |  |  |  |  |  |  |  |  |  |
| Total fat mass, % | 8 | 29.3 | ± | 3.3 |  | 7 | 34.6 | ± | 12.4 | 0.58 |
| Total lean mass, kg | 8 | 49.4 | ± | 3.8 |  | 7 | 44.8 | ± | 1.9 | 0.53 |
| ALM, kg | 8 | 21.8 | ± | 2.0 |  | 7 | 19.5 | ± | 1.0 | 0.58 |
| ALMI, kg/m^2 †^ | 8 | 7.4 | ± | 0.4 |  | 7 | 7.3 | ± | 0.4 | 0.78 |
| ALMI Z-scores | 8 | 0.2 | ± | 0.2 |  | 4 | 0.7 | ± | 0.6 | 0.46 |
| BMD, g/cm^2^ | 8 | 1.131 | ± | 0.020 |  | 7 | 1.079 | ± | 0.106 | 0.23 |
| Z-scores^2^ | 8 | -0.1 | ± | 0.3 |  | 7 | -0.5 | ± | 0.4 | 0.15 |
| > -1, n |  | 6 | | |  |  | 6 | | | -- |
| Between -1 and -2, n |  | 1 | | |  |  | 1 | | |  |
| Low for age (< -2), n |  | 1 | | |  |  | 1 | | |  |
| **Spine L1-L4** |  |  |  |  |  |  |  |  |  |  |
| BMD, g/cm^2^ | 8 | 1.140 | ± | 0.030 |  | 7 | 1.079 | ± | 0.067 | 0.28 |
| Z-scores | 8 | -0.5 | ± | 0.4 |  | 7 | -1.0 | ± | 0.5 | 0.27 |
| > -1, n |  | 6 | | |  |  | 5 | | | -- |
| Between -1 and -2, n |  | 1 | | |  |  | 2 | | |  |
| Low for age (< -2), n |  | 1 | | |  |  | 1 | | |  |
| Trabecular Bone Score | 8 | 1.374 | ± | 0.024 |  | 7 | 1.410 | ± | 0.033 | 0.52 |
| **Total Femur^3^** |  |  |  |  |  |  |  |  |  |  |
| BMD, g/cm^2^ | 7 | 1.0 | ± | 0.03 |  | 5 | 1.1 | ± | 0.1 | 0.58 |
| Z-scores^†^ | 7 | -0.1 | ± | 0.4 |  | 5 | 0.1 | ± | 0.5 | 0.86 |
| > -1, n |  | 6 | | |  |  | 4 | | | -- |
| Between -1 and -2, n |  | 1 | | |  |  | 1 | | |  |
| Low for age (< -2), n |  | 0 | | |  |  | 0 | | |  |
| **Femoral Neck** |  |  |  |  |  |  |  |  |  |  |
| BMD, g/cm^2^ | 7 | 1.0 | ± | 0.02 |  | 5 | 1.0 | ± | 0.04 | 0.69 |
| Z-scores^†^ | 7 | -0.3 | ± | 0.3 |  | 5 | -0.3 | ± | 0.3 | 0.65 |
| > -1, n |  | 6 | | |  |  | 4 | | | -- |
| Between -1 and -2, n |  | 1 | | |  |  | 1 | | |  |
| Low for age (< -2), n |  | 0 | | |  |  | 0 | | |  |
| **Femoral Trochanter** |  |  |  |  |  |  |  |  |  |  |
| BMD, g/cm^2^ | 7 | 0.8 | ± | 0.02 |  | 5 | 0.9 | ± | 0.1 | 0.61 |
| Z-scores^†^ | 7 | -0.1 | ± | 0.4 |  | 5 | 0.1 | ± | 0.5 | 0.63 |
| > -1, n |  | 5 | | |  |  | 4 | | | -- |
| Between -1 and -2, n |  | 1 | | |  |  | 1 | | |  |
| Low for age (< -2), n |  | 1 | | |  |  | 0 | | |  |

^1^Values were obtained at the time of DXA scan completion, n =15. Statistical analysis included ANOVA with main effects for sex and genotype (classical or variant PKU). Two of 15 participants were diagnosed with low BMD-for-age, based on Z-scores < -2.

^2^ One participant, whose Z-scores were included in this analysis, required T-scores for interpretation of DXA scan data due to post-menopausal status.

^3^BMD and Z-scores for femur-related DXA data represent an average for 11 of 12 subjects. Three participants have missing DXA data for the femur. Femur data for 1 participant is based on one femur due to presence of metal in the left hip.

† ALMI or ALM/ht^2^ was calculated as the sum of lean mass of arms and legs (kg) / height^2^ (m^2^) [28].

ALM, appendicular lean mass; ALMI, appendicular lean mass index; BMD, bone mineral density; PKU, phenylketonuria.
